# Isolation and Characterization of Magnetic Oil Palm Empty Fruits Bunch Cellulose Nanofiber Composite as a Bio-Sorbent for Cu(II) and Cr(VI) Removal

**DOI:** 10.3390/polym13010112

**Published:** 2020-12-30

**Authors:** Aina Mardhia Khalid, Md. Sohrab Hossain, Norli Ismail, Nor Afifah Khalil, Venugopal Balakrishnan, Muzafar Zulkifli, Ahmad Naim Ahmad Yahaya

**Affiliations:** 1 School of Industrial Technology, Universiti Sains Malaysia, Gelugor, Penang 11800, Malaysia; mardhiakhalid@gmail.com (A.M.K.); norlii@usm.my (N.I.); 2 University of Kuala Lumpur-Malaysian Institute Chemical & Bioengineering Technology (UniKL-MICET), Lot 1988, Taboh Naning, Alor Gajah, Melaka 78000, Malaysia; muzafar@unikl.edu.my (M.Z.); ahmadnaim@unikl.edu.my (A.N.A.Y.); 3 Institute for Research in Molecular Medicine (INFORMM), Universiti Sains Malaysia, Gelugor, Penang 11800, Malaysia; venugopal@usm.my

**Keywords:** magnetic bio-sorbent, heavy metals adsorption, cellulose nanofiber, isotherm modelling, kinetics studies

## Abstract

In the present study, magnetic oil palm empty fruits bunch cellulose nanofiber (M-OPEFB-CNF) composite was isolated by sol-gel method using cellulose nanofiber (CNF) obtained from oil palm empty fruits bunch (OPEFB) and Fe_3_O_4_ as magnetite. Several analytical methods were utilized to characterize the mechanical, chemical, thermal, and morphological properties of the isolated CNF and M-OPEFB-CNF. Subsequently, the isolated M-OPEFB-CNF composite was utilized for the adsorption of Cr(VI) and Cu(II) from aqueous solution with varying parameters, such as pH, adsorbent doses, treatment time, and temperature. Results showed that the M-OPEFB-CNF as an effective bio-sorbent for the removal of Cu(II) and Cr(VI) from aqueous solution. The adsorption isotherm modeling revealed that the Freundlich equation better describes the adsorption of Cu(II) and Cr(VI) on M-OPEFB-CNF composite. The kinetics studies revealed the pseudo-second-order kinetics model was a better-described kinetics model for the removal of Cu(II) and Cr(VI) using M-OPEFB-CNF composite as bio-sorbent. The findings of the present study showed that the M-OPEFB-CNF composite has the potential to be utilized as a bio-sorbent for heavy metals removal.

## 1. Introduction

Heavy metal contamination in the aquatic environment poses severe environmental pollution concerns worldwide. The primary sources of heavy metals in the aquatic environment are industrial activities such as electroplating, tannery, mining operation, battery manufacturing process, pigment and paint production, and glass manufacturing industry [1,2]. Although some heavy metals in a micronutrient concentration are essential for many biological activities, heavy metals in higher concentrations can cause toxicity effects due to their non-biodegradable nature and accumulation tendency with living organisms [2]. Hexavalent chromium [Cr(VI)] is a toxic heavy metal ion. It is mutagenic and carcinogenic to living organisms. Cr(VI) is a notorious environmental pollutant. The presence of Cr(VI) in the aquatic environment may pose a severe threat to aquatic plants and animals [3]. Cu(II) has widespread application in manufacturing various products, including building construction materials, household products, electronic products, pharmaceuticals machinery, chemical machinery, various alloys, dry cell batteries, and automotive parts [2,4]. However, Cu(II) is a toxic element for aquatic organisms. An excessive amount of Cu(II) in the aquatic environment poses a life threat to the fish, algae, and invertebrates [1,4]. Therefore, removing heavy metals from the aquatic environment is crucial to preserve human health and the environment.

Various technologies, including adsorption [5], precipitation [6], flotation [6,7] electroflotation [8,9], ion exchange [10], solvent extraction [4], and membrane filtration [11], are utilized for the separation of heavy metals from industrial effluent. Among these techniques, adsorption is considered a promising method for removing heavy metal from effluent because of its distinct advantages over other technologies, including easy to operate, cost-effective, and element trace metal ions [5,12,13,14]. Various materials such as clay [12], activated carbon [13], chelating materials [14], and natural polymer [15] have been utilized as an adsorbent for the elimination of the heavy metals from industrial effluents. However, the low sorption efficiency of these adsorbents has limited their application in the adsorption of heavy metals [2,3]. An ideal adsorbent for removing heavy metals from industrial effluent contains a large surface area, high selectivity, mechanical stability, easy accessibility, biodegradable, cost-effectiveness, and environmentally friendly [3,16].

There is increasing interest of utilizing biobased materials from the natural resources in various industrial and environmental applications due to the rapid depletion of global petrochemical-based resources and environmental pollution concern [15,17]. Among the various biobased materials, the plant cellulosic materials have attracted wide interest because of environmentally friendly nature, abundance availability, biodegradability and renewability [17,18]. Le Phuong et al. [17] utilized bamboo fiber as a renewable material in the fabrication of the sustainable and biodegradable nonwoven composite membrane. Abdul Karim et al. [18] utilized banana fiber obtained from the banana leaves as bio-sorbent for the removal of dye from the wastewater. Wang et al. [19] fabricated superhydrophobic coating and sorbent using hydrophobic cellulose nanofiber suspension for oil-water separation. Cellulose nanofiber (CNF) isolated from lignocellulosic biomass has been extensively utilized as an adsorbent, because of its biocompatibility, high porosity, high surface area, abundance availability, non-toxicity, reusability and biodegradability [16,20,21]. Besides, the presence of primary and secondary hydroxyl groups in the carbon chain makes the CNF a promising adsorbent for the elimination of heavy metals from industrial effluent [16]. Studies reported that poor chemical resistance and weak mechanical strength are the significant barriers to use CNF as an adsorbent for the removal of heavy metals from industrial effluent [3,14]. Moreover, it requires expensive technology like the membrane filtration process to separate the CNF after the adsorption process [2].

Recently, magnetic bio-sorbents have received considerable attention to environmental scientists to eliminate heavy metals from industrial effluents [16,22,23]. The distinct advantages of the magnetic bio-sorbent include low cost, mechanical and thermal strength, non-toxic nature, and environmentally friendly [22,23]. Besides, the magnetic bio-sorbent can be easily separated using a magnet after the adsorption process. Thus, an engineering conversion of CNF into a magnetic bio-sorbent would eliminate the existing limitation of CNF to be used as a promising bio-sorbent. Peng et al. [2] utilized the magnetic cellulose-chitosan composite as a bio-sorbent to remove Cu(II) from an aqueous solution. The study reported that the synthesized magnetic cellulose-chitosan composite could be utilized as a potential bio-sorbent because of its biocompatibility, large surface area, porous structure, and affinity adsorb Cu(II) [2]. Sun et al. [3] reported that the magnetic cellulose nanocomposite is the promising adsorbent for eliminating Cr(VI) because of its high adsorption ability, rapid adsorption rate, and easy regeneration of the bio-sorbent under a magnetic field.

It is essential to have consistent raw materials supply at large industrial-scale operations in the practical engineering application. As heavy metal contamination in the aquatic environment poses serious environmental pollution concerns, the development of bio-based materials (biosorbent) has gained attention for the greener elimination of heavy metals from industrial effluents [2,4]. The utilization of the lignocellulosic biomass as a bio-sorbent is attractive because of the abundance availability, low cost, biodegradable, and reusable [3,16]. Generally, the lignocellulosic biomass mainly consists of cellulose, hemicellulose, and lignin with high hydroxyl groups and other reactive functional groups on the surface [16,21]. There is increasing interest in using cellulose nanofiber (CNF) as a bio-sorbent in wastewater treatment because of its large surface area, smaller particle size distribution, high porosity, excellent mechanical and thermal properties [18,24,25]. The CNF has been isolated from the various lignocellulosic biomass, including bamboo [24], rice straw [25], sugarcane bagasse [26] cotton [27], and oil palm biomass [28]. Malaysia is the second-largest palm oil producer and exporter country. It is being reported that the palm oil industries in Malaysia are generating over 40 million tonnes of oil palm biomass, including about 23 million tons of oil palm empty fruits bunches [28,29]. The high cellulose content of OPEFB (≥44%) and its huge generation make the OPEFB ideal for producing CNF to use as a bio-sorbent [29]. Over the years, various methods have been implemented to isolate the cellulose from various lignocellulosic biomasses, including chemical process, mechanical process, and chemo-mechanical process [27,28,30]. Among these various processes, the acid hydrolyses process has been extensively utilized to produce CNF from the various lignocellulosic fibers [28,30] H_2_SO_4_ or HCl are the most common inorganic acids used in the acid hydrolysis process to remove amorphous regions for producing CNF. However, Fahma et al. [31] reported that the acid hydrolysis using H_2_SO_4_ provided the most stable suspension due to the presence of the sulphate group on the surface of the crystallites.

In the present study, a magnetic cellulose nanofiber composite was fabricated using nanocellulose isolated from the oil palm empty fruits bunch (OPEFB). Wherein the Fe_3_O_4_ was utilized as magnetite to coat the surface of the CNF. Several analytical methods were implemented to characterize the fabricated magnetic oil palm cellulose nanofiber (M-OPEFB-CNF) to determine its usability in heavy metals adsorption. Subsequently, M-OPEFB-CNF composite was utilized for the absorption of Cr(VI) and Cu(II) from aqueous solution. Moreover, the adsorption isotherm and kinetics behavior were assessed by fitting the experimental data with several theoretical models.

## 2. Materials and Methods

### 2.1. Materials

The OPEFB collected from Sime Darby Plantation Sdn Bhd (Carey Island, Malaysia) was utilized for cellulose nanofiber isolation. 1-Butyl-3methylimidazolim chloride (analytical grade, purity ≥ 99%) was purchased from Sigma Aldrich (St. Louis, MO, USA). Ferric chloride hexahydrate (FeCl_3_·6H_2_O; analytical grade, purity ≥ 98%), cupric sulphate pentahydrate (CuSO_4_·5H_2_O; analytical grade, purity ≥ 98%), potassium dichromate (K_2_Cr_2_O_7_; analytical grade, purity ≥ 99%) were purchased from Merck (Subang Jaya, Malaysia). All other chemicals used were in analytical grade.

### 2.2. Isolation of Cellulose Nanofiber

The isolation of cellulose nanofiber from the OPEFB was conducted following the method reported by Fatah et al. [30]. The OPEFB collected from the Sime Darby Plantation was cut to 2–3 cm and dried in the open air before proceeding with the soda-pulping process. Approximately 78 g of sodium hydroxide (NaOH) pellets were taken into 300 mL of deionized water to produce cooking liquor. Subsequently, the ground OPEFB and cooking liquor were poured into the digester with a ratio of 1:10 and treated at 161 °C for 10 min and then neutralized the pulp by washing with deionized water. The neutralized pulped were then mechanically disintegrated in a three-bladed disintegrator for 1 min with 2% consistency and screened on a 0.15 mm slits flat-plate screen. The pulped OP-EFB was then bleached using hydrogen peroxide to remove the lignin, hemicelluloses further, and extractives from the OP-EFB. The bleaching process was conducted with OP-EFB to hydrogen peroxide ratio of 1:2, pH 10.5, temperature 70 °C, and treatment time 90 min, wherein the pH of the bleached slurry was adjusted using 1 M sodium hydroxide solution. After bleaching, the slurry was washed with deionized to neutralize and dried using an oven at 60 °C for 24 h.

**Figure 1:**
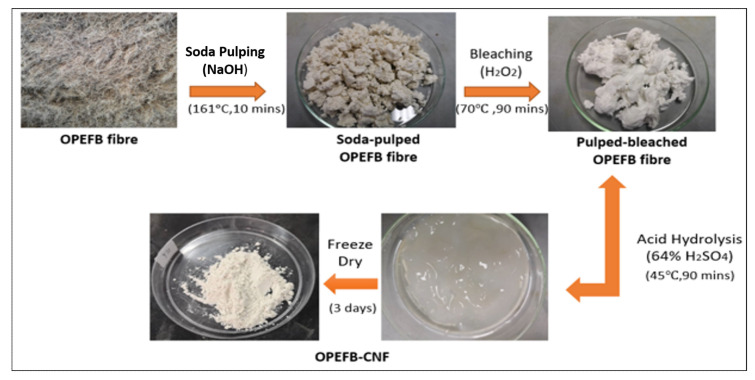
Isolation of cellulose nanofiber from oil palm empty fruits bunch.

Acid hydrolysis was conducted to remove the amorphous regions of bleached OP-EFB to obtain the CNF. 20 g dried bleached OP-EFB were hydrolyzed in 64 wt% sulphuric acid solution at 45 °C for 90 min. The acid hydrolyses process was interrupted by adding 400 mL cold distilled water. The dilute suspension was then centrifuged at 10,000 rpm for 10 min to gain precipitate. The precipitate was collected and taken into a cellulose membrane dialysis tube for dialysis using deionized water. It was tied both ends of the dialysis tube with thread and dialyzed by changing deionized water until obtained the neutral pH (pH 7). It was estimated that it requires 10 mL of water for washing per gram CNF. Subsequently, the OP-EFB nanofiber was sonicated at 50% amplitude for 30 min to disperse nanofibers. The nitrocellulose suspension was then freeze-dried for 3 days to obtain the powder form of nanocellulose. The schematic diagram for the isolation of CNF from OPEFB is shown in Figure 1.

### 2.3. Preparation of Magnetic OP-EFB Nanofiber Composite

**Figure 2:**
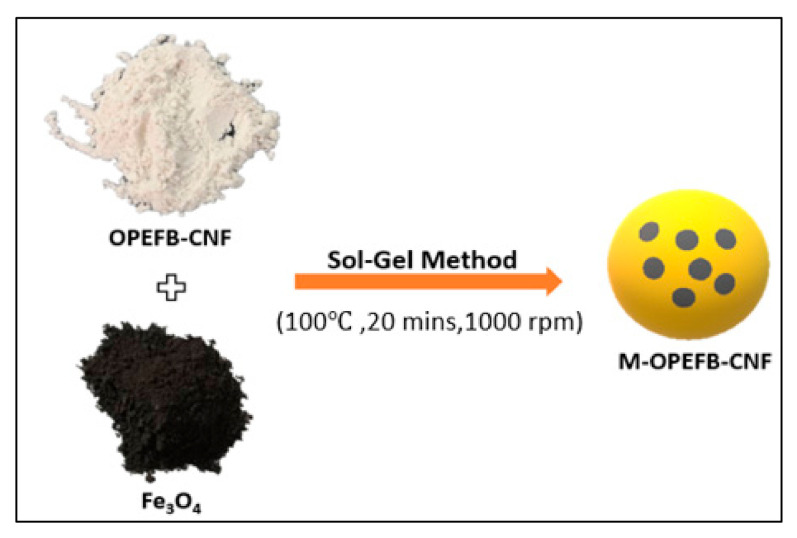
Fabrication of the M-OPEFB-CNF composite by sol-gel method.

The magnetic OP-EFB nanofiber composite’s isolation was conducted using Fe_3_O_4_ as magnetite, as shown in Figure 2. Firstly, the preparation of the Fe_3_O_4_ was carried out using the sol-gel method [2]. 2 g of FeCl_3_·6H_2_O and 8 g of sodium acetate were taken into 80 mL of ethylene glycol. The solution was stirred vigorously using a magnetic stirrer until a clear solution formed. After dissolved, the solution was heated to 100 °C for 16 h to form the dark brown gel. The dark brown gel was washed with ethanol and distilled water and eventually centrifuged at 4000 rpm for 30 min. The precipitate was collected and dried in an oven at 60 °C for 5 h, followed by drying in a furnace at 300 °C for 15 min.

The preparation of magnetic OP-EFB cellulose nanofiber (M-OPEFB-CNF) composite was adapted from the study conducted by Liu et al. [32]. 5 g of CNF was dissolved in 1-butyl-3-methylimidazolium chloride at 100 °C for 30 min. The dissolved CNF was magnetized by adding synthesized magnetic Fe_3_O_4_ powder, and the mixture was vigorously agitated for 20 min using a magnetic stirrer. Subsequently, the well-mixed solution was emulsified by adding 80 mL vacuum pump oil and 4 mL Tween 80, and string at string speed of 1000 rpm in a 100 °C oil bath. The mixture was then cooled to room temperature at a cooling rate of 5 °C per 10 min, while ethanol was added dropwise into the mixture to regenerate the M-OPEFB-CNF composite further. The M-OPEFB-CNF was decanted from the oil suspension and washed several times with ethanol and distilled water to remove the residuals. Finally, the M-OPEFB-CNF composite was dried in a vacuum oven at 50 °C for 3 h and stored at 4 °C before further utilization.

### 2.4. Characterization

The morphology and structural changes of the raw OP-EFB fiber, soda-pulped fiber, bleached pulps, CNF, Fe_3_O_4,_ and M-OPEFB-CNF were analyzed using scanning electron microscopy (SEM-Model: Quanta FEG 650). The samples were ground into powder form and dried in an oven at 50 °C overnight before proceeding to SEM imaging. Transmission Electron Microscopy (TEM-Model: Zeiss Libra 120) was also used to analyze the morphology and nanostructure of the OP-EFB CNF. Fourier transform infrared spectroscopy (FT-IR-Model: SHIMAZU IRPrestige-21 Spectrophotometer) was used to determine the changing of functional groups and chemical bonding of Raw OP-EFB, soda-pulped fibers, bleached pulps, nanocellulose, Fe_3_O_4_ and M-OPEFB-CNF composite. The samples were prepared by following the KBr-disk method. The frequency range of the spectrophotometer was between 4000–400 cm^−1^. The determination of the crystallinity and phase purity of the CNF, Fe_3_O_4_ and M-OPEFB-CNF composite was conducted using X-ray diffraction (XRD) at ambient temperature (28 ± 1 °C) with Cu Kα radiation at 40 kv and 40 mA. The crystallinity index (CI) of the OPEFB-CNF was determined using the Equation below [19].

(1) $$CI\left(\%\right)=\frac{I_{200}-I_{am}}{I_{200}}\times 100$$

where *I*_200_ represents the intensity of amorphous and crystalline regions, and *I_am_* represents the intensity at the amorphous region. Thermal gravimetric analysis (TGA) (Model: PerkinElmer Pyris 1 TGA) was used to determine the thermal stability of the CNF, Fe_3_O_4_ and M-OPEFB-CNF. About 10 mg of sample was placed on the pan and heated with nitrogen gas purge from 30 °C to 700 °C at a heating rate of 10 °C per min.

### 2.5. Adsorption of Cr(VI) and Cu(II) Using M-OPEFB-CNF Composite

Adsorption of Cr (VI) and Cu(II) was carried out using M-OPEFB-CNF with varying pH (pH-1 to pH 8), doses (0.05 gL^−1^ to 1.0 gL^−1^), temperature (28 °C–80 °C) and treatment time (15 min–90 min). A certain amount of adsorbent was taken in 100 mL glass conical flask containing 50 mL of metals ion solution. The M-OPEFB-CNF and the metals ion solution were then mixed using a magnetic stirrer under vigorous agitation. The pH of the aqueous solution was adjusted by adding concentrated H_2_SO_4_ and NaOH solution. After adsorption, the M-OPEFB-CNF was separated using a magnet, and the metals ion concentration of the eluent was determined by the atomic absorption spectrophotometer (AAS) (Model: SHIMADZU AA-7000 AAS). The percentage removal of Cr(VI) and CU(II) was determined using the following Equation.

(2) $$\mathrm{Removal}\;\left(\%\right)\;=\frac{C_{i}{-C}_{t}}{C_{i}}\times 100$$

where, $C_{i}$ represents the initial concentration of metals ion (mgL^−1^), and $C_{t}$ represents the concentration of metals ion (mgL^−1^) after the adsorption process at a time. Wherein, the metals ion uptake capacity at equilibrium (*q_e_*) was calculated using the following Equation.

(3) $$q_{e}=\frac{C_{i}-C_{e}}{D}\times V$$

where *Ce* represents the metal ion concentration at equilibrium, *D* is the doses (mg) of the M-OPEFB-CNF composite, and *V* is the volume (L) of the aqueous phase. All the experiments are conducted in triplicate, and the data presented as the mean value ± standard deviation from the triplicate experimental runs.

### 2.6. Adsorption Isotherm

In the present study, Langmuir and Freundlich isotherm models were used to fit the experimental data. The adsorption experiments were carried out with varying dosage (0.05 gL^−1^ to 0.5 gL^−1^) as a function of the adsorption time (5 min to 90 min) at pH 5 and ambient temperature. The linear regression model was utilized to predict the best-fitted model to describe adsorption behavior for the removal of Cu(II) and Cr(VI) from aqueous solution using M-OPEFB-CNF as adsorbent. The Freundlich isotherm equation and the linear form of the Freundlich isotherm equation can be written, as shown in Equations (4) and (5).

(4) $$q_{e}=K_{f}C_{e}^{\frac{1}{n}}$$

(5) $$\log q_{e}=\log K_{f}+\frac{1}{n}\log C_{e}$$

where, $K_{f}$ is the Freundlich affinity coefficient (Lmg^−1^), while n is the Freundlich exponential constant. The Langmuir isotherm model equation and its linear form can be expressed, as shown in Equations (6) and (7).

(6) $$q_{e}=\frac{abC_{e}}{1+aC_{e}}$$

(7) $$\frac{1}{q_{e}}=\frac{1}{abC_{e}}+\frac{1}{b}$$

where a is the Langmuir constant and b the optimal adsorption value for the removal of Cu(II) and Cr(VI) from aqueous solution using M-OPEFB-CNF as adsorbent. The *b* values for the Langmuir isotherm model was utilized to predict the adsorption process for the removal of Cu (II), and Cr (VI) from aqueous solution using M-OPEFB-CNF composite as adsorbent is favorable or unfavorable.

### 2.7. Kinetics Modelling

Pseudo-first-order and pseudo-second-order kinetic model equations were utilized to ascertain the kinetics behavior of M-OPEFB-CNF for the removal of Cu(II) and Cr(VI) from aqueous solution. The experiments were conducted with varying temperature from ambient temperature (28 ± 1 °C to 70 °C) as a function of the adsorption time (5 min to 90 min) at pH 5, adsorbent dose of 25 mg in 50 mL aqueous solution containing Cu (II) of 200 ppm and Cr (VI) of 100 ppm. The pseudo-first-order kinetic model equation can be written as below [26].

(8) $$\ln(q_{e}-q_{t})=\ln q_{e}-k_{1}t$$

where $q_{e}$ and $q_{t}$ represents the capability of Cu(II) and Cr(VI) adsorption at equilibrium and at time *t* (min), respectively; $k_{1}$ represents the pseudo-first-order rate constant (min^−1^). The pseudo-second-order equation can be written, as shown in Equation (9) [20,26].

(9) $$\frac{t}{q_{t}}=\frac{1}{k_{2}q_{e}^{2}}+\frac{t}{q_{e}}$$

where $k_{2}$ (mg mg^−1^min^−1^) represents the pseudo-second-order adsorption rate constant.

### 2.8. Reusability

The reusability of the M-OPEFB-CNF composite was determined by the adsorption and desorption of Cu(II) for five cycles. At first, the M-OPEFB-CNF composite was used adsorbed Cu(II) from 50 mL aqueous solution containing 200 ppm Cu(II) at pH 5, doses 0.5 gL^−1^, adsorption time of 30 min and at ambient temperature. After adsorption, the M-OPEFB-CNF composite was separated using a magnet, and the Cu(II) loaded M-OPEFB-CNF composite was taken into 50 mL 0.5 M HCl solution. The mixture was then shaken using a mechanical shaker at 150 rpm for 1 h. The M-OPEFB-CNF composite was removed from the acid solution, and the eluent was taken to determine the desorbed Cu(II) into the solution. For the sorbent reuse, the M-OPEFB-CNF composite washed with the deionized water and reused for the next adsorption/desorption cycles.

## 3. Result and Discussion

### 3.1. Morphological Observation of OP-EFB Cellulose and M-OPEFB-CNF

**Figure 3:**
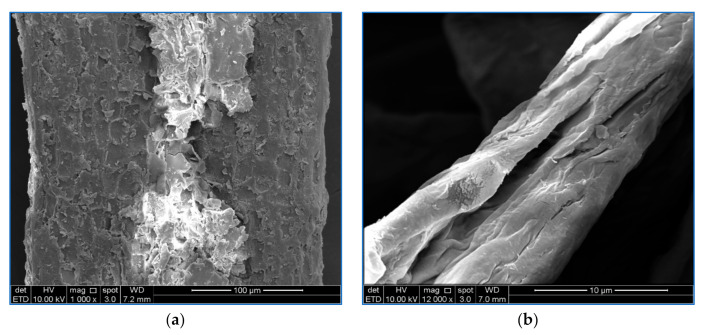

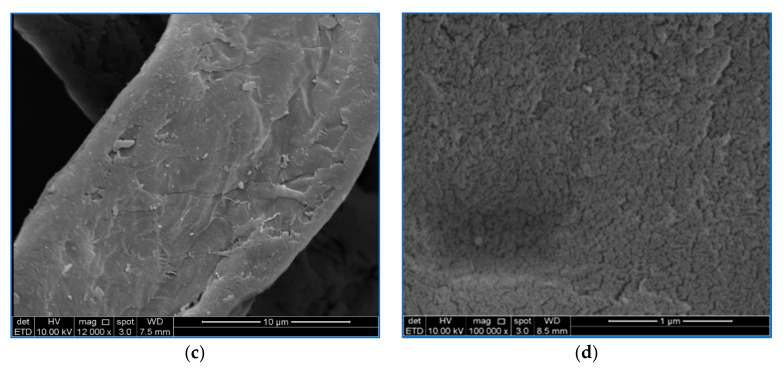
SEM images of (**a**) raw OP-EFB, (**b**) pulped OP-EFB fiber, (**c**) bleached OP-EFB fiber, and (**d**) OPEFB-CNF.

Figure 3 shows the surface morphology of raw OP-EFB fiber, pulped OP-EFB fiber, bleached OP-EFB fiber, and cellulose nanofiber obtained from OP-EFB fiber. It was found that the surface of the SEM image of OP-EFB fiber (Figure 3a) is uneven and rough. This is because of the surface of OP-EFB is covered with non-cellulosic components such as lignin, hemicelluloses and other impurities like wax and oil [27]. The surface of the soda pulping fibers (Figure 3b) become smooth, and the surface roughness reduced due to the removal of impurities by the sodium hydroxide interactions, which replace the hydrogen bonding with OH-groups in the cellulosic hydroxyl groups [24,27]. However, the pulped OP-EFB fiber further improved with bleaching using H_2_O_2_ as a bleaching agent. It was found that the surface morphology of bleached OP-EFB fiber become cleaner and smoother (Figure 3c). This is due to the non-cellulosic fibres such as lignin and hemicelluloses as well as impurities present in the pulped OP-EFB fibres were further reduced, broken down and solubilized during the bleaching process. The bleached fibers were further treated with concentrated sulphuric acid to reduce the amorphous region of the fibres. It was observed that the fibers’ size reduces to nano size, but the surface morphology of the fibres remained unchanged [30]. Similarly, Fahma et al. [31] reported that the acid hydrolysis process reduced the fiber size by changing the surface morphology of the fibres.

**Figure 4:**
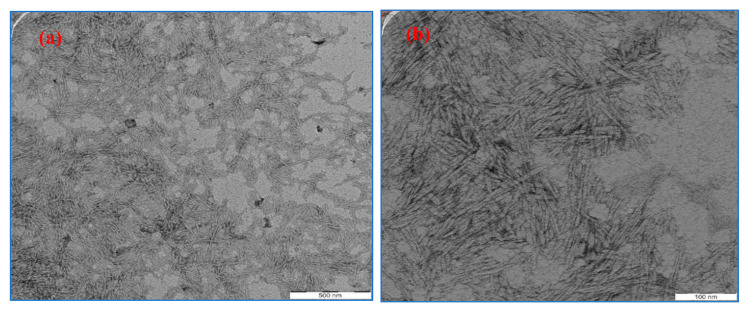
TEM micrographs of OPEFB-CNF. (**a**) 50,000× magnification; (**b**) 100,000× magnification.

Transmission electron microscope (TEM) images of OPEFB-CNF is shown in Figure 4. As can see in TEM images, the isolated OPEFB-CNF is an individually nanofibriled cellulose fiber with little agglomeration. The range of OPEFB-CNF diameter was determined to be 8–15 nm. Several studies isolated cellulose nanofiber from the OPEFB, and the size of OPEFB-CNF has been reported to be 5–40 nm [26,28,29,30]. Fatah et al. [30] isolated OPEFB-CNF using the chemo-mechanical technique. The study observed that the isolated OPEFB-CNF was agglomerated nanofibriled fiber with a 5–10 nm fiber diameter. Liu et al. [32] reported that the agglomeration of the isolated OPEFB-CNF was due to the surface ionic charge (sulfate ion and hydrogen ion) during the acid hydrolysis process.

**Figure 5:**
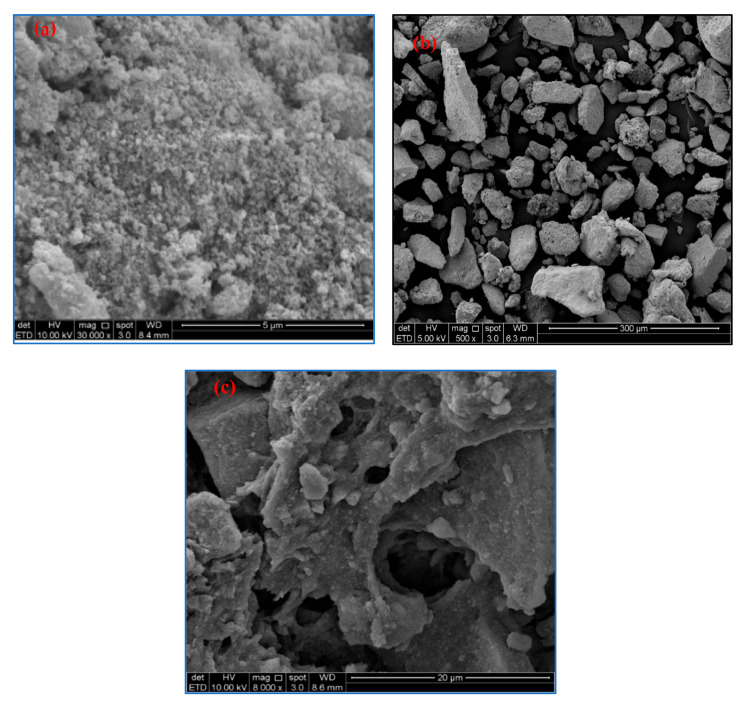
SEM images of (**a**) Fe_3_O_4_, (**b**) M-OPEFB-CNF composites, and (**c**) M-OPEFB-CNF composites at magnification: 8000×.

Figure 5 shows the SEM images of Fe_3_O_4_ and fabricated M-OPEFB-CNF composite. As can see in Figure 5a, the SEM image of the Fe_3_O_4_ was agglomerated. The agglomeration of the Fe_3_O_4_ was due to the high surface energy and Van der Waals force of inter-particles [32,33]. The size of the Fe_3_O_4_ was observed to be nanosized with a diameter range of 100–200 nm. The surface morphology of M-OPEFB-CNF composites appears to be rough and irregular in shape (Figure 5b). This was due to the aggregation between the particles due to the combination of Fe_3_O_4_ and CNF [3]. However, there is a porous structure found on the surface of M-OPEFB-CNF composites. Studies reported that the higher the porosity of the materials, the higher the surface area, and therefore, the material has a high adsorption ability [22,34]. Porous materials are promising adsorbent for the removal of heavy metal because of heaving high surface area. Thus, the porosity structure of the isolated M-OPEFB-CNF composites has the potential to be used as an adsorbent to remove heavy metals.

### 3.2. FT-IR Analyses

**Figure 6:**
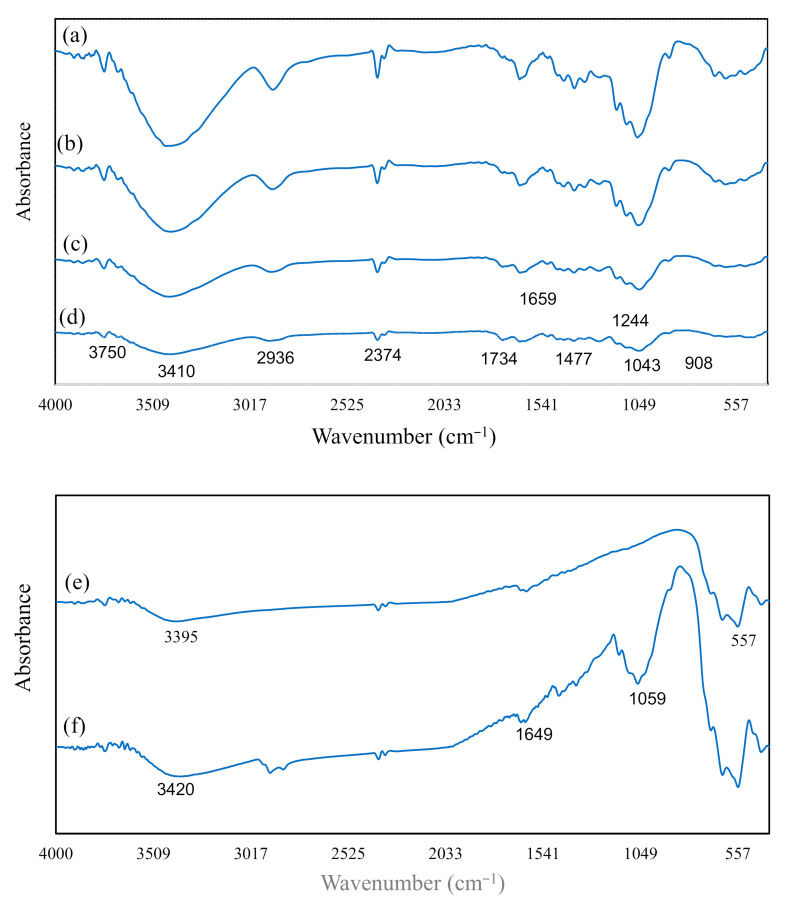
FT-IR spectra of (**a**) Raw OPEFB fiber, (**b**) pulped OPEFB fiber, (**c**) bleached-pulped OPEFB fiber, (**d**) CNF, (**e**) Fe_3_O_4_, (**f**) M-OPEFB-CNF.

The chemical composition changes of OPEFB fiber because of pulping with NaOH (Figure 6b), bleaching with H_2_O_2_ (Figure 6c) and acid hydrolysis with H_2_SO_4_ (Figure 6d) were determined using FT-IR spectroscopy analyses, as shown in Figure 6. There are two main regions of absorbance peaks observed for OPEFB fiber, pulped OPEFB fiber, bleached OPEFB fiber, and CNF. The first absorbance peak appeared in the wavenumber range of 2800–3500 cm^−1^, whereas the second region was between 800–1750 cm^−1^ wavenumber. The appearance of the similar absorbance peaks in the FT-IR spectra for OPEFB fiber, pulped OPEFB fiber, bleached OPEFB fiber, and CNF indicated that the alkaline, bleaching and acid hydrolysis treatments had not affected the chemical structure of the fibers [27]. The peak at 3420–3424 cm^−1^ represents the stretching hydroxyl groups (O-H bonds). This is due to the moisture content found in the non-cellulosic fibers, which give them the hydrophilic property [35]. The presence of 2911 cm^−1^ absorption band shows the stretching vibration of -CH_2_ in cellulose, hemicellulose, and lignin [36]. Moreover, the absorption peak at 1038–1051 cm^−1^ represents the C-O-C stretching vibration, which is the pyranose rings present in cellulose [37].

The absorption peak of 1647–1655 cm^−1^ (O-H stretching) was detected in the FT-IR spectra of pulped OPEFB fiber, bleached OPEFB fiber and CNF, and this peak was absent in the raw OPEFB fiber spectra. This indicated that alkaline treatment with sodium hydroxide had caused water molecules’ formation due to the reaction between sodium hydroxide and the hydroxide groups in cellulose [38]. The presence of 1460 cm^−1^ and 1238 cm^−1^ absorption bands on raw OPEFB spectra represents the methoxyl-O-CH_3_ and C-O-C bond (aryl-alkyl ether) in lignin [38]. These peaks were absent in pulped OPEFB fiber, bleached OPEFB fiber, and CNF spectra, indicated that the lignin was removed during alkaline and bleaching treatment. The absorbance peak at the 1740 cm^−1^ the C=O bonds in ketone and carbonyl groups in hemicellulose. However, the absence of the peak at 1740 cm^−1^ in CNF spectra reveals no hemicellulose present in the nanocellulose because of alkaline and bleaching treatments of the OPEFB fiber [30]. The absorbance peak at 887 cm^−1^ shows the apparent presence of β-glycosidic linkages of glucose ring in cellulose. As shown in Figure 6, the absorbance peak intensity at 887 cm^−1^ increased gradually along with the alkaline, bleaching, and acid treatments of OPEFB fibers. This indicates that the hemicellulose and lignin had been effectively removed from the OPEFB fibers with alkaline pulping and bleaching process [30,35].

FT-IR spectra of Fe_3_O_4_ and M-OPEFB-CNF are shown in Figure 6. It was found that the O-H stretching vibration presence at absorption bands of 3420 cm^−1^ and 1665 cm^−1^ in M-OPEFB-CNF spectra (Figure 6f). Wherein the O-H stretching vibration presence at absorption bands of 3420 cm^−1^ in Fe_3_O_4_ spectra (Figure 6e). The absorbance peak at 3420 cm^−1^ and 1665 cm^−1^ in M-OPEFB-CNF spectra and CNF (Figure 6e) was due to the water molecules in cellulose. However, the hydroxyl group in Fe_3_O_4_ may be due to the reaction of ethylene glycol, which was part of the materials used to generate this ferric oxide [33]. The absence of the absorption peak at 1061 cm^−1^ in Fe_3_O_4,_ which is the pyranose ring that only present in the cellulose structure. The absorption peak at 555 cm^−1^ presents in both Fe_3_O_4_ and M-OPEFB-CNF spectra attributes to the formation of Fe-O bands [2,33]. This peak was absent in CNF spectra; this indicated that the nanocellulose was successfully combined with Fe_3_O_4_ during the fabrication of M-OPEFB-CNF [33,36].

### 3.3. X-ray Diffraction

**Figure 7:**
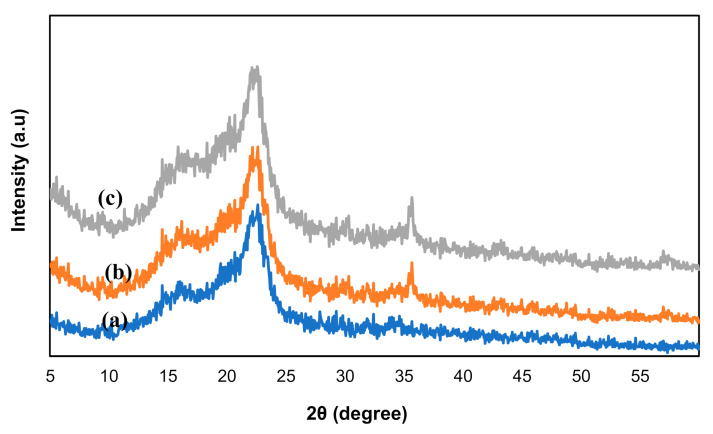
X-ray diffraction analyses for (**a**) CNF, (**b**) Fe_3_O_4_ and (**c**) M-OPEFB-CNF.

Figure 7 shows the X-ray diffraction (XRD) pattern of OPEFB-CNF (Figure 7a), Fe_3_O_4_ (Figure 7b) and M-OPEFB-CNF (Figure 7c). There are two peaks intensity observed in the XRD pattern of OPEFB-CNF pattern (Figure 7a), the broad peak at 16° and the prominent diffraction peak at 22°. The presence of both the peaks in the OPEFB-CNF pattern indicated that the OPEFB-CNF is obtained from the cellulose I type [30,35]. The percentage crystallinity index was calculated to be about 63.34% for the OPEFB-CNF. The XRD peaks at 2*θ* value of 22°, 30.2°, 35.6°, 43.3° and 57.5° were observed on the XRD patterns of Fe_3_O_4_ and M-OPEFB-CNF. Similarly, Peng et al. [2] also observed the XRD peaks at 22°, 30.2°, 35.6°, 43.3° and 57.5° on the XRD results of Fe_3_O_4_. These peaks on the XRD patterns indicated the spinel structure of crystalline magnetite [2,33]. Since the OPEFB-CNF had similar peaks as in Fe_3_O_4_, it can be concluded that the Fe_3_O_4_ attached with OPEFB-CNF.

### 3.4. Thermal Gravimetric Analysis

**Figure 8:**
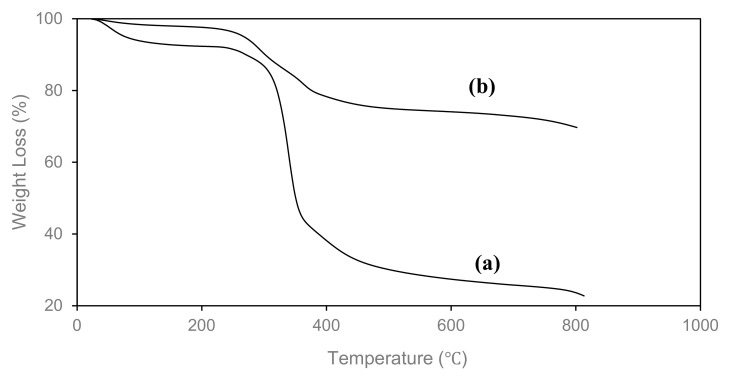
TGA analysis of (**a**) OPEFB-CNF and (**b**) M-OPEFB-CNF.

Thermogravimetric analyses (TGA) were carried out to study the thermal stability of the OPEFB-CNF, Fe_3_O_4,_ and M-OPEFB-CNF composite, as shown in Figure 8. It was observed that there are two stages of thermal decomposition of the M-OPEFB-CNF, which occurred at 257.2 °C and 351.7 °C. The first decomposition stage from 25 °C to 257.2 °C was due to the dehydration process in the M-OPEFB-CNF composite [35]. The decomposition of the M-OPEFB-CNF composite from 257.2 °C to 351.7 °C was due to the degradation of iron [36]. The onset decomposition temperature of OPEFB-CNF was determined to be 316.7 °C. The M-OPEFB-CNF composite decomposed at a lower temperature than OPEFB-CNF was due to the weaker hydrogen bonding (intramolecular and intermolecular) M-OPEFB-CNF composite as the result of Fe_3_O_4_ incorporation [39]. Similarly, Peng et al. [16] observed that the onset decomposition temperature of magnetic chitosan-cellulose microspheres was lower than the pure cellulose. However, the residual masses of the M-OPEFB-CNF composite after decomposition was higher than the OPEFB-CNF, indicating that the Fe_3_O_4_ particles have been embedded with the M-OPEFB-CNF composite. The finding of TGA analyses implies that the Fe_3_O_4_ has successfully bonded with the M-OPEFB-CNF composite, which is also in agreement with the finding of FT-IR and XRD analyses.

### 3.5. Adsorption of Cr(VI) and Cu(II)

**Figure 9:**
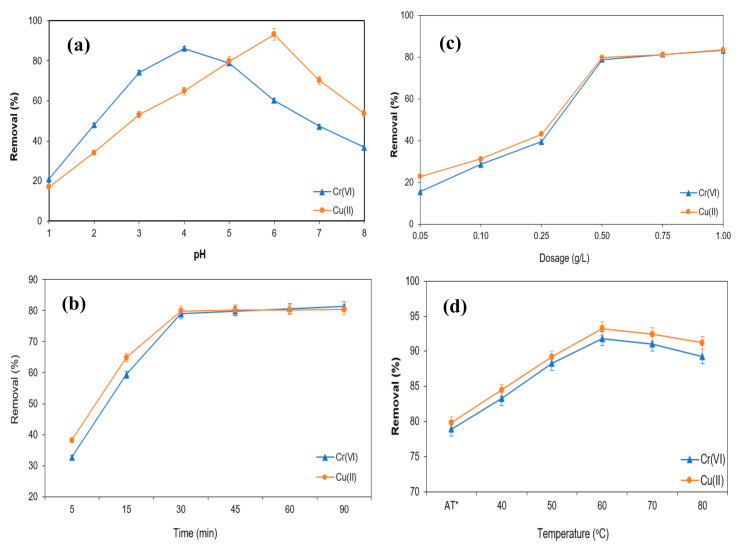
Removal of Cr(VI) and Cu(II) from aqueous solution using M-OPEFB-CNF composite as a bio-sorbent. (**a**) Effect of pH, (**b**) Effect of doses (gL^−1^), (**c**) Effect of adsorption time (min), (**d**) Effect of Temperature (°C).

One of the essential variables in the adsorption process is the pH of the metal ion solution since pH influences both the ionization state of the functional groups and the chemical speciation present in the adsorbent [40]. The initial influence pH of the aqueous solution on the removal of Cr(VI) and Cu(II) was determined using M-OPEFB-CNF as a bio-sorbent with varying pH at doses 0.5 gL^−1^, treatment time 30 min and at ambient temperature (28 ± 1 °C), as shown in Figure 9a. It was found that the percentage removal of Cu(II) and Cr(VI) was increased with increasing pH up to pH 4 and pH 6 for the removal of Cr(VI) and Cu(II), respectively, and decrease thereafter with further increasing of the pH of the aqueous solution. The lowest percentage of Cu (II) and Cr (VI) removal obtained at pH 1 (17.14% and 21.07%, respectively), the maximum removal of Cu(II) and Cr(VI) obtained were 93.23% and 86.24% at pH 6 and pH 4, respectively.

pH is the most critical parameter for the heavy metal adsorption using a bio-sorbent. It influences the bio-sorbent transition in removing heavy metals by modifying the reactive functional group of the bio-sorbent. At low pH, the removal of Cu(II) and Cr(VI) was lower because of the competition of bio-sorbent reactive sites with positive ions, resulting in the minimal adsorption of Cu(II) and Cr(VI). With an increase in the pH of the aqueous solution, the surface charge of the M-OPEFB-CNF becomes negative due to the deprotonation of the reactive functional group of M-OPEFB-CNF composite. This enhances the electrostatic interaction between metal ions and bio-sorbent, increasing Cu(II) and Cr(VI) adsorption efficiency. Over the optimal pH for Cu(II) and Cr(VI) removal (pH 6 and pH 4, respectively), the increase of pH of the aqueous solution leads to the protonation, result in electric repulsion between M-OPEFB-CNF composite and metals ion and hence decrease the removal of Cu(II) and Cr(VI). Based on the study, it has been postulated that the removal of Cr(VI) and Cu(II) using M-OPEFB-CNF composite as a bio-sorbent is pH-dependent. Similarly, Ahmad et al. [40], Vishnu et al. [41] and Daneshfozoun et al. [42] reported that the removal of heavy metals subjected to magnetic bio-sorbent is pH dependent. Daneshfozoun et al. [42] found that the maximum recovery of Cu(II) was 92.2% at pH 6 using oil palm fiber-based magnetic bio-sorbent.

The influence of adsorbent doses on the removal of Cu(II) and Cr(VI) from aqueous solution using M-OPEFB-CNF composite was determined with varying adsorbent doses (0.05 g L^−1^–1 g L^−1^) at pH 5, treatment time of 30 min, and metals ion concentration of 200 ppm and 100 ppm for Cu(II) and Cr(II), respectively. The results obtained are presented in Figure 9b. It was observed that the percentage removal of Cu(II) and Cr(VI) concentration increased with increasing the M-OPEFB-CNF composite doses up to 0.5 g L^−1^, thereafter the removal efficiency of Cu(II) and Cr(VI) decreased with further increasing of the M-OPEFB-CNF composite doses. The highest Cu(II) and Cr(VI) removal obtained were 79.83% and 78.95%, respectively. The increase of the Cu(II) and Cr(VI) removal with the adsorbent doses because of increasing active functional groups for the adsorption. Thus, as the dosage increased, the ratio between the numbers of adsorption sites to the number of heavy metal ions would increase, and un-adsorbed adsorption sites would be abundant. Nevertheless, the percentage removal of metal ion removal achieved a saturated value at a dosage of 0.5 g L^−1^. The decrease of the Cu(II) and Cr(VI) removal with the increasing the M-OPEFB-CNF composite doses over 0.5 g L^−1^ might occur due to the saturation active adsorption site for binding Cu(II) and Cr(VI) [43]. Another possible cause could be particle aggregation, which leads to a decrease in the total surface area of the adsorbent [44].

Figure 9c shows the influence of adsorption time on the removal of Cu(II) and Cr(VI) from aqueous solution using M-OPEFB-CNF composite as a bio-sorbent. The experiments were conducted with varying adsorption time from 5 min to 90 min at constant pH 5, M-OPEFB-CNF composite doses of 0.5 g L^−1^, and ambient temperature. It was found that the removal of Cu(II) and Cr(VI) increased rapidly with increasing adsorption time from 5 min to 30 min. Ahead of 30 min adsorption time, the increase of Cu(II) and Cr(VI) removal from the aqueous solution was negligible. The negligible increase of Cu(II) and Cr(VI) removal was observed ahead of 30 min adsorption time might due to the saturation of the surface of the M-OPEFB-CNF composite with Cu(II) and Cr(VI). Therefore, the metal ion binding affinity on the M-OPEFB-CNF composite surface diminished after 30 min adsorption time, which may decline the Cr(VI) and Cu(II) adsorption efficiency. However, The removal of Cr(VI) and Cu(II) gained at 30 min adsorption time about 79% and 80%, respectively. Similarly, Vishnu et al. [41] reported that 30 min adsorption time is the optimal time for the removal of Cu(II) and Cr(VI) using magnetic microspheres of *Muraya koenigii* extract.

The influences of temperature on the removal of Cu(II) and Cr(VI) from the aqueous solution using M-OPEFB-CNF composite as a bio-sorbent was determined as presented in Figure 9d. The experiment was conducted with varying temperatures from ambient temperature (28 ± 1 °C) to 80 °C at pH 5.0, M-OPEFB-CNF composite doses of 0.5 g L^−1^, and an adsorption time of 30 min. It was found that the removal of the Cr(VI) and Cu(II) from aqueous solution using M-OPEFB-CNF composite as a bio-sorbent increased with increasing temperature up to 60 °C and slightly decreased with the further increase of the temperature. The highest Cu(II) and Cr(VI) removal obtained at 60 °C temperature were 93.25% and 91.82%, respectively. The increase of the Cu(II) and Cr(VI) removal with increasing temperature might due to the busting of internal bonds at a higher temperature, resulting in increased active sites on the surface of the M-OPEFB-CNF composite. Conversely, the kinetics of Cu(II) and Cr(VI) increased with increasing temperature, enhancing the affinity of binding the bio-sorbent and, therefore, increasing the adsorption efficiency. Similarly, Fan et al. [45] reported the adsorption of Cd(II), Zn(II), and Pb(II) using *Penicillium simplicissimum* increased with increasing temperature because of the increasing active site on the surface of the adsorbent at the higher temperature. However, both Cu(II) and Cr(VI) have shown similar adsorption behavior with M-OPEFB-CNF composite doses, treatment time, and temperature. This is because both Cu(II) and Cr(VI) are transitional metals and had a similar affinity to bind with the adsorbent under the controlled pH (pH 5) in the aqueous solution.

### 3.6. Adsorption Equilibrium Studies

Equilibrium adsorption isotherms study for removing metals ion from aqueous is vital to understand the adsorption behavior between adsorbent and metals ion present in the aqueous phase [45]. Many sorption isotherms models have been successfully applied to experimental data to determine the adsorption behavior assuming that every adsorbent site is independent for adsorbing the metals ion whether or not the adjacent sites are occupied [13,46]. The adsorption equilibrium studies show the dependence of the metal ion adsorbed on the surface of the adsorbent to conduct the adsorption process. In the present study, Langmuir and Freundlich isotherm models were utilized to describe the adsorption behavior of Cu (II) and Cr (VI) from aqueous solution using M-OPEFB-CNF as a bio-sorbent.

**Figure 10:**
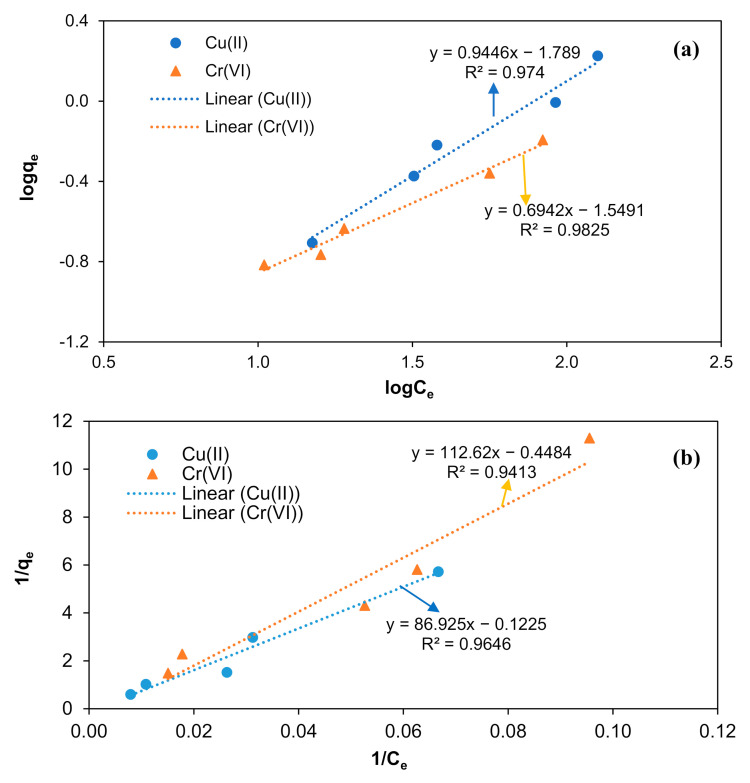
Isotherm modeling for the removal of Cu(II) and Cr(VI) from aqueous solution using M-OPEFB-CNF as a bio-sorbent. (**a**) Freundlich isotherm model, and (**b**) Langmuir isotherm model.

Freundlich isotherm model expresses the adsorption of heavy metals on the surface of the bio-sorbent occurs on the heterogeneous surface of adsorbent in non-uniform distribution and multilayer formation. Nevertheless, the Freundlich isotherm model does not limit the monolayer formation of the adsorption process, and therefore, the adsorption process can be reversible [17,47]. The adsorption of the behavior of Cu(II) and Cr(VI) from aqueous solution using M-OPEFB-CNF as a bio-sorbent was determined using the Freundlich (a) and Langmuir (b) isotherm models, as shown in Figure 10. The Freundlich affinity constant (*K_f_*) value and the Freundlich exponential constant *(n*) values calculated using the Equation (5) for the removal of Cu(II) and Cr(VI) using M-OPEFB-CNF as a bio-sorbent are presented in Table 1. It was found that *K_f_* values for the removal of Cu(II) and Cr(VI) were 0.0162 L mg^−1^ and 0.0282 L mg^−1^, respectively. Wherein the Freundlich exponential constant *(n*) values for the removal of Cu(II) and Cr(VI) were 1.0586 and 1.4405, respectively. The Freundlich exponential constant values obtained were close to 1, indicating that the removal of Cu(II) and Cr(VI) from aqueous solution using M-OPEFB-CNF composite as a biosorbent is favorable [13,46].

Langmuir isotherm model explains the adsorption process occurs in uniform distribution of adsorbate molecules in monolayer formation and homogeneous distribution [47]. The Langmuir isotherm model was originally developed to describe the gas adsorption behavior on the surface of activated carbon. However, the isotherm model has been extensively used to describe adsorption behavior and quantify the adsorption with various bio-sorbent. Figure 10b demonstrates the Langmuir isotherm for the removal of Cu(II) and Cr (VI) using M-OPEFB-CNF as a bio-sorbent. It was found that Langmuir constant (*a*) values for the removal of Cu(II) and Cr(VI) were −0.0014 Lmg^−1^ and −0.0034 Lmg^−1^, respectively. Besides, the maximum adsorption values (*b*) values for the removal of Cu(II) and Cr(VI) were −81633 mg mg^−1^ and −2.2301 mg mg^−1^, respectively. The calculated Langmuir constant (*a*) values and maximum adsorption (*b*) values were negative, indicating that the adsorption of Cu(II) and Cr(VI) using M-OPEFB-CNF does not comply with the Langmuir isotherm concept [43]. Therefore, the possible adsorption behavior for the removal of Cr(VI) and Cu(II) from aqueous solution using M-OPEFB-CNF as a bio-sorbent is the multilayer formation with non-homogeneous distribution (Freundlich isotherm) [45,48].

Table 1 shows the coefficient of determination (*R^2^*) values of Freundlich and Langmuir’s isotherms modeling for the removal of Cu(II) and Cr(VI) using M-OPEFB-CNF as a bio-sorbent. The *R^2^* values of Freundlich isotherm were 0.9740 and 0.9825 for the removal of Cu(II) and Cr(VI), respectively. The *R^2^* values of Freundlich isotherm were 0.9646 and 0.9413 for the removal of Cu(II) and Cr(VI), respectively. It was found that the predicted *R^2^* values of the Freundlich isotherm model greater than the predicted *R^2^* values of the Langmuir isotherm model (Table 1). The greater *R^2^* values of Freundlich isotherm modeling indicate that the Freundlich isotherm is the better-fitted model for describing the adsorption behavior of Cu(II) and Cr(VI) using M-OPEFB-CNF as a bio-sorbent. Besides, the negative Langmuir constant (*a*) values and maximum adsorption (*b*) values also indicate the Freundlich isotherm is the best-described model for the removal of Cr(VI) and Cu(II) from aqueous solution using M-OPEFB-CNF as a bio-sorbent. Similarly, Alijerf [49] stated that the Freundlich isotherm was the best-described isotherm model for the adsorption of heavy metals using zeolite as an adsorbent. Badawi et al. [50] described that the Freundlich isotherm was the best-fitted isotherm model to describe Al(III) and Pb(II) adsorption behavior using tannin as a bio-sorbent.

### 3.7. Adsorption Kinetics

**Figure 11:**
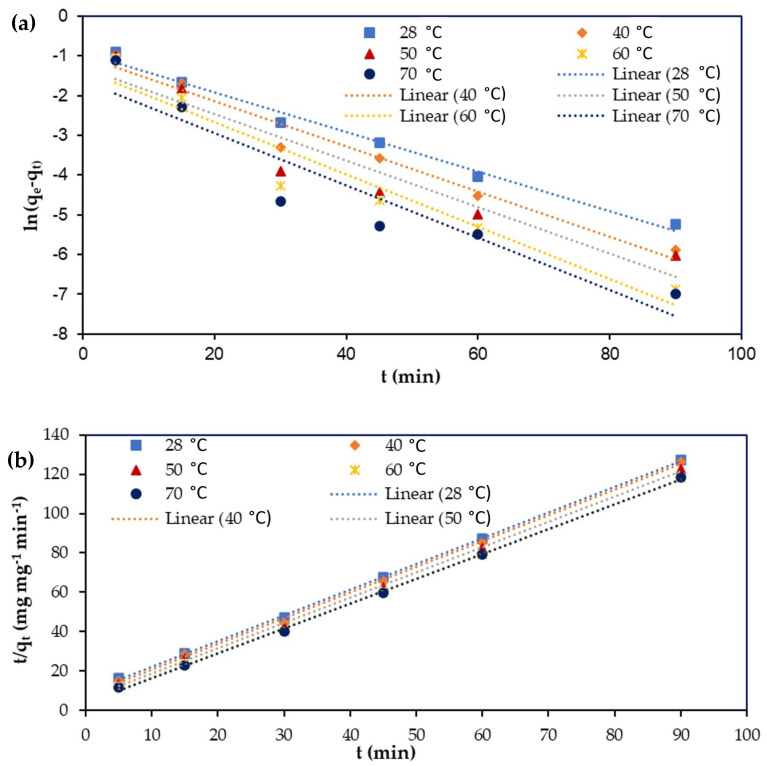
Adsorption kinetics modeling for the removal of Cu(II)) from aqueous solution using M-OPEFB-CNF as a bio-sorbent. (**a**) Pseudo-first-order kinetic model, and (**b**) pseudo-second-order kinetic model.

**Figure 12:**
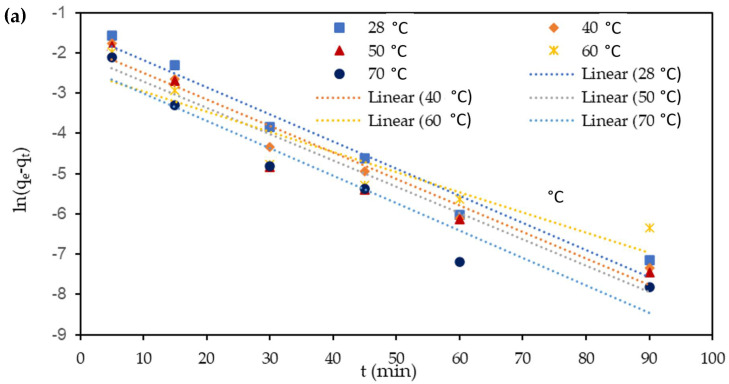

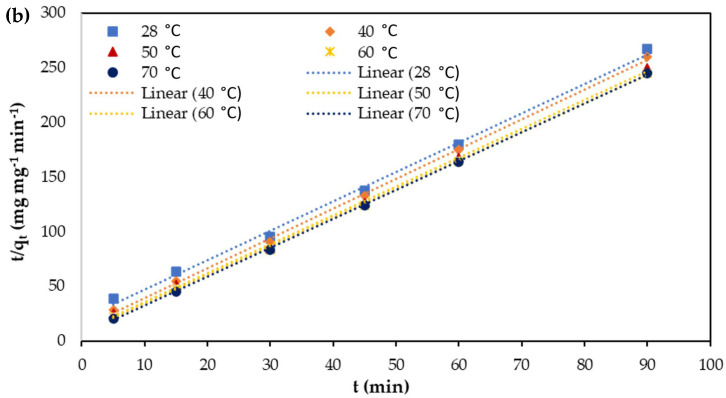
Adsorption kinetics s modeling for the removal of Cu(II) and Cr(VI) from aqueous solution using M-OPEFB-CNF as a bio-sorbent. (**a**) Pseudo-first-order kinetic model and (**b**) pseudo-second-order kinetic model.

The determination of the adsorption kinetics is crucial to determine the adsorption behavior. Generally, the adsorption kinetics depends on the mass transport process and the chemical features of the adsorbent [45,51]. In the present study, pseudo-first-order and pseudo-second-order kinetic models were utilized to predict the adsorption kinetics for the removal of Cu(II) and Cr(VI) from aqueous solution using M-OPEFB-CNF composite as a bio-sorbent. The adsorption kinetics for the removal of Cu(II) and Cr(VI) from aqueous solution using M-OPEFB-CNF as a bio-sorbent were determined as shown in Figure 11 and Figure 12, respectively. The adsorption capacity implied by *q_t_* (mg mg^−1^) at each predetermined time interval was obtained from the kinetic study. The uptake capacity at the equilibrium time interval is represented as *q_e_* (mg mg^−1^). The adsorption kinetics is used to determine the rate of the uptake of organic particles, which describes the uptake capacity of adsorbate on the surface of the adsorbent at each equilibrium contact time. The plot of ln (*q_e_ − q_t_*) against t (min) was used to determine the pseudo-first-order kinetics for the removal of Cu(II) and Cr(VI) from aqueous solution using M-OPEFB-CNF as a bio-sorbent. The values of *q_e_* (mg mg^−1^) and *k_1_* were determined from the slope and intercept of the linearized plot of the pseudo-first-order kinetic model [37]. A slope of $\frac{1}{qe}$ and the intercept $\frac{1}{K2}$ *qe*^2^ represents the graph of $\frac{t}{qt}$ against *t* (min) for the pseudo-second-order kinetic models.

Table 2 shows the predicted pseudo-first-order rate constant (*k_1_*) and pseudo-second-order rate constant (*k_2_*), q_e,_ and *R^2^* values for the removal of Cu(II) and Cr(VI) from aqueous solution using M-OPEFB-CNF composite as a bio-sorbent. The variations between experimental and predicted *q_e_* values and correlation coefficients were employed to identify the best-fitted kinetics model for the removal of Cu(II) and Cr(VI) from aqueous solution using M-OPEFB-CNF as a bio-sorbent. It was found that the *R^2^* values for the pseudo-second-order kinetic model (R^2^ > 0.999) were closer to unity than the pseudo-first-order kinetic model (R^2^ < 0.999). Furthermore, the values of experimental *q_e_* for Cu(II) and Cr(VI) were more closely matched with the theoretical *q_e_* values of the pseudo-second-order kinetic model compared to those from the pseudo-first-order kinetic model. Thus, we inferred that the adsorption mechanism for the removal of Cu(II) and Cr(VI) from aqueous solution using M-OPEFB-CNF as a bio-sorbent was better described by the pseudo-second-order kinetic model. Similarly, Chen et al. [52] found that the pseudo-second-order kinetic model was well-fitted kinetics model for the removal of Ni(II) and Cu(II) using treated alga. Vishnu et al. [41] reported that the pseudo-second-order kinetic model was the best-fitted kinetics model for the removal of Cu(II) and Cr(VI) using magnetic *Muraya koeniggi* extract.

### 3.8. Reusability of the M-OPEFB-CNF Composite as a Biosorbent

**Figure 13:**
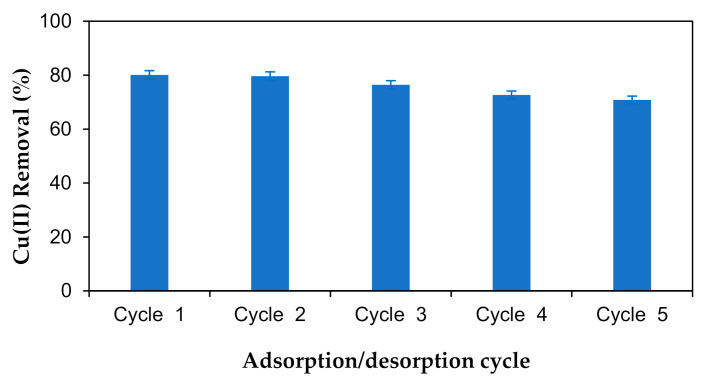
Determination of the reusability potential of M-OPEFB-CNF composite as a biosorbent for Cu(II) removal.

The reusability of the M-OPEFB-CNF composite in the adsorption of heavy metals was determined by adsorption of Cu(II) from the aqueous solution and desorption using HCl solution. The adsorption and desorption studies were conducted for five cycles, as shown in Figure 13. It was found that the Cu(II) adsorption efficiency slightly decreased with the adsorption cycles. About 80% Cu(II) removal efficiency was gained at adsorption cycle 1, which is reduced to about 71% at absorption cycle 5. Thus, it can be postulated that the M-OPEFB-CNF composite has the potential for reusability, thus confirming the distinct advantage of magnetic biosorbent for the separation of heavy metals from industrial effluent. Similarly, Daneshfozoun et al. [42] reported that the magnetic biosorbent has good metal removal efficiency with the potential of reusability and recoverability. Naushad [53] observed that the surfactant assisted nano-composite cation exchanger had good metal ion uptake and separation efficiency.

Based on the finding of the present study, the isolated M-OPEFB-CNF composite is an effective bio-sorbent for the removal of Cu(II) and Cr(VI) from aqueous solution. Besides, the reusability of the M-OPEFB-CNF composite makes it a promising material for utilizing in heavy metals removal from industrial effluent. Although the M-OPEFB-CNF composite has the potential to be used as a promising adsorbent for the heavy metals removal from industrial effluent, the preparation method is time-consuming. Besides, the application of the 1-butyl-3-methylimidazolium chloride as an ionic liquid makes the fabrication process of M-OPEFB-CNF composite costly. Therefore, the present study suggests determining an alternative ionic liquid of the 1-butyl-3-methylimidazolium chloride to make the M-OPEFB-CNF composite fabrication process economical. Besides, the present study urges to further studies to assess the environmental impact and economic analyses before implementing large-scale operation for the industrial effluents treatment using M-OPEFB-CNF composite as a biosorbent.

## 4. Conclusions

In the present study, M-OPEFB-CNF composite was fabricated by sol-gel method using ionic liquid as a solvent for the absorption of Cr(VI) and Cu(II) from the aqueous solution. The CNF was successfully isolated from OPEFB using the acid hydrolysis process. The surface morphology of M-OPEFB-CNF composites exhibits porous structure and irregular in shape. Analyses of FT-IR, XRD, and TGA revealed that the Fe_3_O_4_ has successfully bonded with the OPEFB-CNF. It was found that pH, adsorbent doses, treatment time, and the temperature had influenced the Cu(II) and Cr(VI) removal. The maximum removal of Cu(II) and Cr(VI) obtained were 93.25% and 91.82%, at pH 5 M-OPEFB-CNF composite dose of 0.5 g L^−1^, treatment time of 30 min, and at temperature 60 °C. The Freundlich equation was the better-fitted isothermal model for describing the adsorption behavior, revealing that the removal of Cu(II) and Cr(VI) occurred non-uniform distribution and multilayer formation. The pseudo-second-order kinetic model was the better-fitted kinetics model for the removal of Cu(II) and Cr(VI) using M-OPEFB-CNF composite as a bio-sorbent. Determination of the reusability of M-OPEFB-CNF composite shows that the M-OPEFB-CNF composite had the potential for reusability for the adsorption of metal ions, confirming the distinct advantage of magnetic biosorbent for heavy metal ion adsorption. In conclusion, the M-OPEFB-CNF composite could be utilized as a promising adsorbent for removing heavy metals, including Cu(II) and Cr(VI) from the industrial effluent. However, it urges to conduct further studies to assess the environmental impact and economic analyses before implementing large-scale operation to remove heavy metals from industrial effluents using M-OPEFB-CNF composite as a biosorbent.

## Figures and Tables

**Table 1:** Freundlich and Langmuir model for the removal of Cu(II) and Cr(VI) from aqueous solution using M-OPEFB-CNF as a bio-sorbent.

Adsorbate	Freundlich Model			Langmuir Model		
	*R^2^*	*K_f_* (L mg^−1^)	n	*R^2^*	a (L mg^−1^)	b(mg mg^−1^)
Cu(II)	0.9740	0.0162	1.0586	0.9646	−0.0014	−8.1633
Cr (VI)	0.9825	0.0282	1.4405	0.9413	−0.0034	−2.2301

**Table 2:** Kinetics parameters for the removal of Cu(II) and Cr(VI) from aqueous solution using M-OPEFB-CNF as a bio-sorbent.

Parameters	Temperature (°C)	q_e_ (exp) (mg mg^−1^)	Pseudo-First-Order Kinetics			Pseudo-Second Order Kinetics		
			q_e_ (mg mg^−1^)	k_1_ (min^−1^)	*R^2^*	q_e_ (mg mg^−1^)	K_2_ (mg mg^−1^ min^−1^)	*R^2^*
Cu(II)	28	0.7069	0.4012	0.1152	0.9725	0.7349	0.1901	0.9996
	40	0.7127	0.3678	0.1310	0.9669	0.7646	0.2287	0.9988
	50	0.7337	0.2722	0.1350	0.9049	0.7793	0.2812	0.9985
	60	0.7599	0.2600	0.1518	0.9302	0.7959	0.3660	0.9991
	70	0.7608	0.1993	0.1541	0.8849	0.7921	0.4323	0.9995
Cr(VI)	28	0.33712	0.2225	0.1552	0.9725	0.3418	0.3583	0.9971
	40	0.34612	0.1588	0.1513	0.9645	0.3670	0.6103	0.9991
	50	0.36104	0.1284	0.1508	0.9322	0.3789	0.7337	0.9991
	60	0.36772	0.0866	0.1154	0.8587	0.3808	0.9561	0.9996
	70	0.36808	0.0988	0.1571	0.9381	0.3785	1.2573	0.9998

## Data Availability

The data presented in this study are available on request from the corresponding author.

## References

[1] Carolin C.F., Kumar P.S., Saravanan A., Joshiba G.J., Naushad M. (2017). Efficient techniques for the removal of toxic heavy metals from aquatic environment: A review. J. Environ. Chem. Eng..

[2] Peng S., Meng H., Ouyang Y., Chang J. (2014). Nanoporous Magnetic Cellulose–Chitosan Composite Microspheres: Preparation, Characterization, and Application for Cu(II) Adsorption. Ind. Eng. Chem. Res..

[3] Sun X., Yang L., Li Q., Zhao J., Li X., Wang X., Liu H. (2014). Amino-functionalized magnetic cellulose nanocomposite as adsorbent for removal of Cr(VI): Synthesis and adsorption studies. Chem. Eng. J..

[4] Bari F., Hossain S., Mujtaba I.M., Jamaluddin S.B., Hussin K. (2009). Simultaneous extraction and separation of Cu(II), Zn(II), Fe(III) and Ni(II) by polystyrene microcapsules coated with Cyanex 272. Hydrometallurgy.

[5] Manirethan V., Raval K., Rajan R., Thaira H., Balakrishnan R.M. (2018). Kinetic and thermodynamic studies on the adsorption of heavy metals from aqueous solution by melanin nanopigment obtained from marine source: Pseudomonas stutzeri. J. Environ. Manag..

[6] Balladares E., Jerez O., Parada F., Baltierra L., Hernandez C., Araneda E., Parra V. (2018). Neutralization and co-precipitation of heavy metals by lime addition to effluent from acid plant in a copper smelter. Miner. Eng..

[7] Zhao W., Liu D., Feng Q. (2020). Enhancement of salicylhydroxamic acid adsorption by Pb(II) modified hemimorphite surfaces and its effect on floatability. Miner. Eng..

[8] Cole M., Dickinson J., Galvin K. (2020). Recovery and cleaning of fine hydrophobic particles using the Reflux™ Flotation Cell. Sep. Purif. Technol..

[9] Selvaraj R., Santhanam M., Selvamani V., Sundaramoorthy S., Sundaram M. (2018). A membrane electroflotation process for recovery of recyclable chromium(III) from tannery spent liquor effluent. J. Hazard. Mater..

[10] Feng Y., Yang S., Xia L., Wang Z., Suo N., Chen H., Long Y., Zhou B., Yu Y. (2019). In-situ ion exchange electrocatalysis biological coupling (i-IEEBC) for simultaneously enhanced degradation of organic pollutants and heavy metals in electroplating wastewater. J. Hazard. Mater..

[11] Efome J.E., Rana D., Matsuura T., Lan C.Q. (2018). Experiment and modeling for flux and permeate concentration of heavy metal ion in adsorptive membrane filtration using a metal-organic framework incorporated nanofibrous membrane. Chem. Eng. J..

[12] Mnasri-Ghnimi S., Frini-Srasra N. (2019). Removal of heavy metals from aqueous solutions by adsorption using single and mixed pillared clays. Appl. Clay Sci..

[13] Shahrokhi-Shahraki R., Benally C., El-Din M.G., Park J. (2021). High efficiency removal of heavy metals using tire-derived activated carbon vs commercial activated carbon: Insights into the adsorption mechanisms. Chemosphere.

[14] Tighadouini S., Radi S., Anannaz M., Bacquet M., Degoutin S., Tillard M., Eddike D., Amhamdi H., Garcia Y. (2018). Engineering β-ketoenol structure functionality in hybrid silica as excellent adsorbent material for removal of heavy metals from water. New J. Chem..

[15] Yao M., Wang Z., Liu Y., Yang G., Chen J. (2019). Preparation of dialdehyde cellulose graftead graphene oxide composite and its adsorption behavior for heavy metals from aqueous solution. Carbohydr. Polym..

[16] Peng S., Liu Y., Xue Z., Yin W., Liang X., Li M., Chang J. (2017). Modified nanoporous magnetic cellulose–chitosan microspheres for efficient removal of Pb(II) and methylene blue from aqueous solution. Cellulose.

[17] Le Phuong H.A., Ayob N.A.I., Blanford C.F., Rawi N.F.M., Szekely G. (2019). Nonwoven Membrane Supports from Renewable Resources: Bamboo Fiber Reinforced Poly(Lactic Acid) Composites. ACS Sustain. Chem. Eng..

[18] Karim S.K.A., Lim S.F., Chua S.N.D., Salleh S.F., Law P.L. (2016). Banana Fibers as Sorbent for Removal of Acid Green Dye from Water. J. Chem..

[19] Wang X., Liu F., Li Y., Zhang W., Bai S., Zheng X., Huan J., Cao G., Yang T., Wang M. (2020). Development of a facile and bi-functional superhydrophobic suspension and its applications in superhydrophobic coatings and aerogels in high-efficiency oil–water separation. Green Chem..

[20] Yasin N.M.F.M., Hossain S., Abdul Khalil H.P.S., Zulkifli M., Al-Gheethi A., Asis A.J., Yahaya A.N.A. (2020). Treatment of Palm Oil Refinery Effluent Using Tannin as a Polymeric Coagulant: Isotherm, Kinetics, and Thermodynamics Analyses. Polymers.

[21] Oyewo O.A., Elemike E.E., Onwudiwe D.C., Onyango M.S. (2020). Metal oxide-cellulose nanocomposites for the removal of toxic metals and dyes from wastewater. Int. J. Biol. Macromol..

[22] Li B., Zhang Q., Pan Y., Li Y., Huang Z., Li M., Xiao H. (2020). Functionalized porous magnetic cellulose/Fe_3_O_4_ beads prepared from ionic liquid for removal of dyes from aqueous solution. Int. J. Biol. Macromol..

[23] Gu H., Zhou X., Lyu S., Pan D., Dong M., Wu S., Ding T., Wei X., Seok I., Wei S. (2020). Magnetic nanocellulose-magnetite aerogel for easy oil adsorption. J. Colloid Interface Sci..

[24] Zhang X., Zhao J., Cheng L., Lu C., Wang Y., He X., Zhang W. (2014). Acrylic acid grafted and acrylic acid/sodium humate grafted bamboo cellulose nanofibers for Cu^2+^adsorption. RSC Adv..

[25] Bisla V., Rattan G., Singhal S., Kaushik A. (2020). Green and novel adsorbent from rice straw extracted cellulose for efficient adsorption of Hg (II) ions in an aqueous medium. Int. J. Biol. Macromol..

[26] Khoo R.Z., Chow W., Ismail H. (2018). Sugarcane bagasse fiber and its cellulose nanocrystals for polymer reinforcement and heavy metal adsorbent: A review. Cellulose.

[27] Abu-Danso E., Srivastava V., Sillanpää M., Bhatnagar A. (2017). Pretreatment assisted synthesis and characterization of cellulose nanocrystals and cellulose nanofibers from absorbent cotton. Int. J. Biol. Macromol..

[28] Septevani A.A., Rifathin A., Sari A.A., Sampora Y., Ariani G.N., Sudiyarmanto, Sondari D. (2020). Oil palm empty fruit bunch-based nanocellulose as a super-adsorbent for water remediation. Carbohydr. Polym..

[29] Padzil F.N.M., Lee S.H., Ainun Z.M.A., Lee C.H., Abdullah L.C. (2020). Potential of Oil Palm Empty Fruit Bunch Resources in Nanocellulose Hydrogel Production for Versatile Applications: A Review. Materials.

[30] Fatah I.Y.A., Khalil H.P.S.A., Hossain S., Aziz A.A., Davoudpour Y., Dungani R., Bhat A. (2014). Exploration of a Chemo-Mechanical Technique for the Isolation of Nanofibrillated Cellulosic Fiber from Oil Palm Empty Fruit Bunch as a Reinforcing Agent in Composites Materials. Polymers.

[31] Fahma F., Iwamoto S., Hori N., Iwata T., Takemura A. (2010). Isolation, preparation, and characterization of nanofibers from oil palm empty-fruit-bunch (OPEFB). Cellulose.

[32] Liu Z., Wang H., Li B., Liu C., Jiang Y., Yu G., Mu X. (2012). Biocompatible magnetic cellulose–chitosan hybrid gel microspheres reconstituted from ionic liquids for enzyme immobilization. J. Mater. Chem..

[33] Takai Z., Mustafa M., Asman S., Sekak K. (2019). Preparation and Characterization of Magnetite (Fe_3_O_4_) nanoparticles By Sol-Gel Method. Int. J. Nanoelectron. Mater..

[34] Ma Y., Wang Z., Xu X., Wang J. (2017). Review on porous nanomaterials for adsorption and photocatalytic conversion of CO_2_. Chin. J. Catal..

[35] Chieng B.W., Lee S.H., Ibrahim N.A., Then Y.Y., Loo Y.Y. (2017). Isolation and Characterization of Cellulose Nanocrystals from Oil Palm Mesocarp Fiber. Polymers.

[36] Jodeh S., Hamed O., Melhem A., Salghi R., Jodeh D., Azzaoui K., Benmassaoud Y., Murtada K. (2018). Magnetic nanocellulose from olive industry solid waste for the effective removal of methylene blue from wastewater. Environ. Sci. Pollut. Res..

[37] Mandal A., Chakrabarty D. (2011). Isolation of nanocellulose from waste sugarcane bagasse (SCB) and its characterization. Carbohydr. Polym..

[38] Nazir M.S., Wahjoedi B.A., Yussof A.W., Abdullah M.A. (2013). Eco-Friendly Extraction and Characterization of Cellulose from Oil Palm Empty Fruit Bunches. Bioresources.

[39] Li W., Liu J., Qiu Y., Li C., Wang W., Yang Y. (2018). Polyethylene glycol modified magnetic nanoparticles for removal of heavy metal ions from aqueous solutions. J. Dispers. Sci. Technol..

[40] Ahmad S.Z.N., Salleh W.N.W., Ismail A.F., Yusof N., Yusop M.Z.M., Aziz F. (2020). Adsorptive removal of heavy metal ions using graphene-based nanomaterials: Toxicity, roles of functional groups and mechanisms. Chemosphere.

[41] Vishnu D., Dhandapani B., Santhyiya K. (2019). The symbiotic effect of integrated Muraya koenigii extract and surface-modified magnetic microspheres—A green bio-sorbent for the removal of Cu(II) and Cr(VI) ions from aqueous solutions. Chem. Eng. Commun..

[42] Daneshfozoun S., Abdullah M., Abdullah B. (2017). Preparation and characterization of magnetic biosorbent based on oil palm empty fruit bunch fibers, cellulose and Ceiba pentandra for heavy metal ions removal. Ind. Crop. Prod..

[43] Kamal M.H.M.A., Azira W.M.K.W.K., Kasmawati M., Haslizaidi Z., Saime W.N.W. (2010). Sequestration of toxic Pb(II) ions by chemically treated rubber (Hevea brasiliensis) leaf powder. J. Environ. Sci..

[44] Liu P., Oksman K., Mathew A.P. (2016). Surface adsorption and self-assembly of Cu(II) ions on TEMPO-oxidized cellulose nanofibers in aqueous media. J. Colloid Interface Sci..

[45] Fan T., Liu Y., Feng B., Zeng G., Yang C., Zhou M., Zhou H., Tan Z., Wang X. (2008). Biosorption of cadmium(II), zinc(II) and lead(II) by Penicillium simplicissimum: Isotherms, kinetics and thermodynamics. J. Hazard. Mater..

[46] Bedin K.C., Souza I.P., Cazetta A.L., Spessato L., Ronix A., Almeida V.C. (2018). CO_2_-spherical activated carbon as a new adsorbent for Methylene Blue removal: Kinetic, equilibrium and thermodynamic studies. J. Mol. Liq..

[47] Ngteni R., Hossain S., Omar A.M., Asis A.J., Tajudin Z. (2020). Kinetics and Isotherm Modeling for the Treatment of Rubber Processing Effluent Using Iron (II) Sulphate Waste as a Coagulant. Water.

[48] Hossain S., Omar F., Asis A.J., Bachmann R., Sarker Z.I., Ab Kadir M.O. (2019). Effective treatment of palm oil mill effluent using FeSO4.7H2O waste from titanium oxide industry: Coagulation adsorption isotherm and kinetics studies. J. Clean. Prod..

[49] Aljerf L. (2018). High-efficiency extraction of bromocresol purple dye and heavy metals as chromium from industrial effluent by adsorption onto a modified surface of zeolite: Kinetics and equilibrium study. J. Environ. Manag..

[50] Badawi M., Negm A., Kana M.A., Hefni H., Moneem M.A. (2017). Adsorption of aluminum and lead from wastewater by chitosan-tannic acid modified biopolymers: Isotherms, kinetics, thermodynamics and process mechanism. Int. J. Biol. Macromol..

[51] Naushad M., Mittal A., Rathore M., Gupta V. (2015). Ion-exchange kinetic studies for Cd(II), Co(II), Cu(II), and Pb(II) metal ions over a composite cation exchanger. Desalin. Water Treat..

[52] Chen Z., Ma W., Han M. (2008). Biosorption of nickel and copper onto treated alga (Undaria pinnatifida): Application of isotherm and kinetic models. J. Hazard. Mater..

[53] Naushad M. (2014). Surfactant assisted nano-composite cation exchanger: Development, characterization and applications for the removal of toxic Pb^2+^ from aqueous medium. Chem. Eng. J..

